# Up close and visible: correcting global presbyopia

**Published:** 2026-03-14

**Authors:** Abigail Steinberg, Elanor Watts, Patricia Elaine Freels

**Affiliations:** 1 Executive Director: Livelihood Impact Fund, Denver, USA.; 2 Research Consultant: Peek Vision, Technical Advisor: LIF Eyeglasses Initiative, and Ophthalmology Resident: NHS Scotland, Glasgow, UK.; 3 Communications: Livelihood Impact Fund, Istanbul, Turkey.

**Few global health challenges can be solved within a decade. We believe presbyopia – age-related near-vision loss – can be.**

**Figure:**
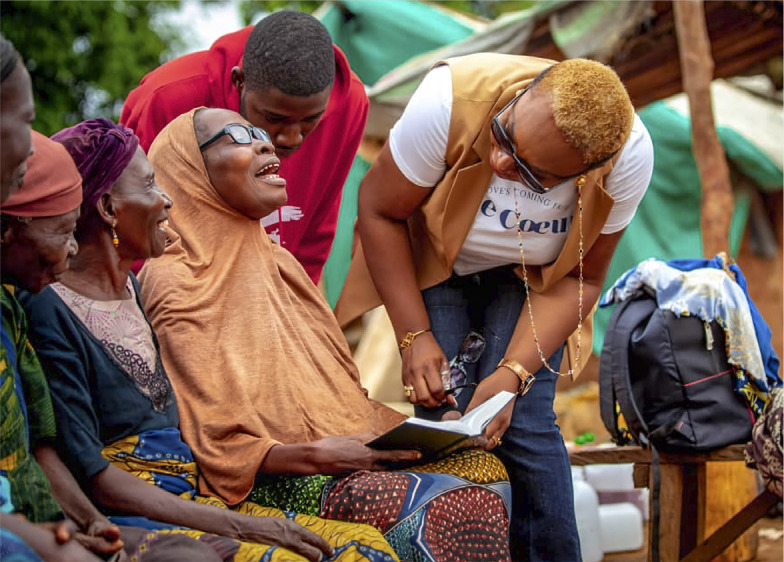
Near-vision spectacles can have a life-changing impact. ZIMBABWE

© PEEK VISION/CBM/CYNTHIA MATONHODZE CC BY-NC-SA 4.0

Presbyopia is an age-related reduction in the eye's ability to focus on near objects (accommodation), and it affects nearly everyone aged 40 years or above. Global estimates are continually being updated;^1^ however, according to the World Health Organization,1.8 billion people have presbyopia, and nearly half of them (as many as 826 million people) do not have access to optical correction (bit.ly/wrvision).

## The benefits of near-vision correction

Historically, eye care services have prioritised distance vision. However, for many of us, near vision may be more important, allowing us to read or write, use our phones, perform self-care tasks like shaving and putting on make-up, or carry out labour-specific tasks such as farming, sewing, carpentry, repair work, or even eye surgery. Without good near vision, life can become very challenging. Fortunately, ready-made near-vision spectacles are a simple and accessible intervention that cost less than USD 1 to manufacture and can dramatically improve vision.

Restored near vision can be transformative. There are benefits to quality of life, adult literacy, autonomy, productivity, income, and empowerment. When a person with presbyopia receives a pair of near-vision spectacles, their ability to take on important tasks again or to learn new skills contributes to an improved sense of dignity, confidence, and self-worth. In one study, women self-reported that near-vision spectacles improved their economic, educational, political, and psychological empowerment.^2^ Presbyopia correction is also a gender equity issue: women comprise the majority of older adults globally and are disproportionately represented in informal work that depends on near vision, yet their coverage is lower than men's.

Studies show that providing spectacles to working people can increase overall productivity by nearly 22%,^2^ rising to 32% in workers over the age of 50 years.^3^ In some settings, this can boost income by up to 33%.^4^ Near-vision impairment from uncorrected or undercorrected presbyopia contributes to an estimated annual productivity loss of USD 25 billion.^5^ Addressing presbyopia with low-cost interventions like near-vision spectacles could yield USD 1.05 trillion in productivity gains by 2050.^6^ These larger-scale economic benefits can prove effective in persuading governments to make changes, and to improve access on a wider scale.

Near vision is now recognised as vital by the World Health Organization (WHO), as set out on pp. 4–5 in this issue. Near vision is included in effective refractive error coverage (near eREC), describing the proportion of those whose near refractive need is met (with good resultant visual acuity). eREC was recently made a WHO Global Health Observatory marker of health coverage. WHO has also launched the SPECS 2030 initiative, aiming to provide high quality, affordable, and people-centred refractive error services to everyone who would benefit. Read more in our previous issue on refractive error: bit.ly/cehj785.

However, access to near-vision spectacles remains limited, especially in low- and middle-income countries. This could be for a number of reasons, including limited access to eye health services, regulatory barriers, social or cultural norms (i.e., around ageing), lack of awareness of presbyopia, or competition with daily survival needs, among others. Removing regulatory barriers, strengthening supply chains, and increasing access points are all essential to support the normalisation and adoption of near-vision spectacles at scale. Philanthropy can play a massive role in supporting this push, as noted on p. 14.

Eye care can struggle to compete for resources within health care, but when viewed through the lenses of development, education, and employment, the investment case for vision is undeniable, especially for a condition that can be managed as affordably as presbyopia. Presbyopia can also be an entry point for strengthening refractive services, primary health care, healthy aging programmes, universal health care, or community health strategies.

Because near-vision spectacles are safe, even people without easy or affordable access to qualified eye care professionals can – and should – be given access to near-vision correction, whether via a primary or community health worker (pp. 6–7), self-selected spectacles purchased over the counter (pp. 8–9) or an innovative distribution strategy (pp. 10–12). If a person finds that their vision does not improve with near-vision spectacles, or they have any other eye health problems, they should be encouraged and supported to visit the nearest eye clinic.

The global burden of uncorrected presbyopia and its social and economic implications on societies demand our attention. Correcting presbyopia may be the most achievable global eye health win of our generation.

## What can we do about this?

**If you're an eye care worker wanting to give your patients access to near-vision spectacles:**

- Remember to check near visual acuity. Some testing options are listed under Useful Resources (right).
- When taking a history, don't skip over presbyopia symptoms or assume the patient already has near-vision spectacles.
- Find out where near-vision spectacles are currently available in your area and consider directing patients to these sources, or collaborate to bring spectacle provision into your workplace.
- Identify those patients who may not be suitable for ready-made spectacles (such as patients with myopia or significant astigmatism), and direct them to optometrists for formal refraction.
- If awareness is the barrier, consider simple awareness raising (such as with the posters included in this issue) or a more intensive campaign.
- Contact your country's national eye care coordinator or do an internet search to find out what projects are running in your country or to ask where you can find affordable near-vision spectacles.

**If you're a policy maker or policy advisor:**

- Support policies that reduce barriers to importing and providing near-vision spectacles, e.g. reducing tariffs.
- Use the WHO Refractive error situational analysis tool to integrate presbyopia into your national eye care strategies (for more, see article on pp. 4–5).
- Support screening and distribution through multiple channels, to ensure everyone has access: over the counter, via existing health channels (e.g. community and primary health workers), and through innovative channels.
- Reach out to and collaborate with organisations and partners already working in your country.
- Unlock funding to support scalable approaches (see philanthropy article on p. 14).
- Consider presbyopia a stepping stone to more comprehensive eye care. Alongside the relatively straightforward task of providing presbyopia correction, refer people for additional care, which improves access to effective distance refractive correction, cataract surgery, and treatment for more complex eye problems.

**Useful resources**

- **WHO Learning on TAP** (bit.ly/4rqkYcu) and WHO's **Vision and eye screening implementation handbook** (bit.ly/49FemAs) make training in vision and eye screening, and dispensing ready-made near-vision spectacles, widely accessible.
- The **WHOeyes app** (bit.ly/4ohzeBu) helps raise awareness about presbyopia and supports efficient screening when paired with an eye health screen.
- **Peek Acuity** (bit.ly/cehj_5DVQ) tools can be used to screen for presbyopia and guide provision of near-vision spectacles.
- **RestoringVision's** partner resources (restoringvision.org/resources) include a tumbling E chart and tips for dispensing; it can be used with their 8-minute training video on dispensing near-vision spectacles (bit.ly/RVnvtraining).
